# Next-Generation Nucleic Acid-Based Diagnostics for Viral Pathogens: Lessons Learned from the SARS-CoV-2 Pandemic

**DOI:** 10.3390/microorganisms13081905

**Published:** 2025-08-15

**Authors:** Amy Papaneri, Guohong Cui, Shih-Heng Chen

**Affiliations:** 1 In Vivo Neurobiology Group, National Institute of Environmental Health Sciences, National Institutes of Health, Research Triangle Park, Durham, NC 27709, USA; amy.papaneri@nih.gov (A.P.); cuig@niehs.nih.gov (G.C.); 2 Viral Vector Core Facility, National Institute of Environmental Health Sciences, National Institutes of Health, Research Triangle Park, Durham, NC 27709, USA

**Keywords:** coronavirus, SARS-CoV-2, epidemiology, detection, transmission, isothermal, CRISPR-Cas, aptasensor, RTPCR, pandemic

## Abstract

The COVID-19 pandemic, caused by Severe Acute Respiratory Syndrome Coronavirus 2 (SARS-CoV-2), catalyzed unprecedented innovation in molecular diagnostics to address critical gaps in rapid pathogen detection. Over the past five years, CRISPR-based systems, isothermal amplification techniques, and portable biosensors have emerged as transformative tools for nucleic acid detection, offering improvements in speed, sensitivity, and point-of-care applicability compared to conventional PCR. While numerous reviews have cataloged the technical specifications of these platforms, a critical gap remains in understanding the strategic and economic hurdles to their real-world implementation. This review provides a forward-looking analysis of the feasibility, scalability, and economic benefits of integrating these next-generation technologies into future pandemic-response pipelines. We synthesize advances in coronavirus-specific diagnostic platforms and attempt to highlight the need for their implementation as a cost-saving measure during surges in clinical demand. We evaluate the feasibility of translating these technologies—particularly CRISPR-Cas integration with recombinase polymerase amplification (RPA)—into robust first-line diagnostic pipelines for novel viral threats. By analyzing the evolution of diagnostic strategies during the COVID-19 era, we aim to provide strategic insights and new directions for developing and deploying effective detection platforms to better confront future viral pandemics.

## 1. Introduction

Since the onset of the global coronavirus disease (COVID-19) pandemic in 2020, Severe Acute Respiratory Syndrome Coronavirus 2 (SARS-CoV-2), the virus that causes COVID-19, has become one of the most extensively investigated viruses of our time. SARS-CoV-2 infections have been quantified on an unparalleled scale over the past five years [1,2,3,4]. Understandably, research for advances in molecular detection of Coronavirinae has been highly prioritized with the aim of being prepared with a more rapid and efficient method of detection for the next novel viral pathogens.

By far, the most common method employed for the detection of SARS-CoV-2 nucleic acids (NA) in clinical samples during the COVID-19 pandemic was the Real-Time Reverse Transcription-Polymerase Chain Reaction (rRT-PCR, RT-qPCR, i.e., RT-PCR). Despite recent biotechnological advancements, traditional RT-PCR has remained the gold standard diagnostic test for many viral pathogen infections [5,6,7,8]. However, this method presents several limitations, which were evident early in the COVID-19 pandemic. RT-PCR requires specialized laboratory equipment, reagents, and skilled personnel, often resulting in turnaround times of up to 72 h from sample collection to results for the conventional method. This delay often hinders the initiation of effective treatment methods. Moreover, RT-PCR deployment in community or low-resource settings is challenging due to limited laboratory infrastructure, trained staff, and cold chain capacity. The higher cost of RT-PCR tests compared to rapid antigen tests and global supply shortages during the pandemic further exacerbated these challenges [9,10,11].

Aside from logistical restraints, efforts to curtail the spread of a viral pathogen are often countered by its antigenic drift and genome recombination rates. These characteristics contribute to the generation of escape mutants: variants that are no longer efficiently neutralized by existing host antibodies [12,13]. For example, coronaviruses are known to have a particularly high recombination rate due to frequent relocation of the RNA-dependent RNA polymerase (RdRP) during transcription [14]. As a result, vaccine development attempts for pathogens such as these “common cold” viruses have historically been recognized as futile [15,16,17]. However, as recent epidemics and pandemics of emerging viral pathogens have caused severe disease, early identification of novel viral threats for the dispensation of effective treatment has proven key to rapid patient recovery and to combating disease spread.

Consequently, the development of superior detection methods has been prioritized, focusing on producing faster, more affordable, and user-friendly approaches, including streamlined sample preparation procedures. This review article summarizes recent advancements, highlighting innovations in viral NA detection, with a particular focus on coronavirus, including CRISPR-Cas-based assays and isothermal amplification methods, which are often combined with rapid biosensors and simplified sample collection. These contemporary tools have the potential to supersede traditional RT-PCR and next-generation sequencing (NGS) as new standards for the identification of coronaviruses and other viral pathogens.

## 2. Coronavirus Epidemiology

Coronaviruses (CoVs), first identified in the 1960s, have remained endemic human pathogens [18,19]. Common human CoVs (HCoVs) are known to cause generally mild respiratory and sometimes enteric disease, including those from the *Alphacoronavirus* genus, such as HCoV-229E and HCoV-NL63 (identified in 2004), and the *Betacoronavirus* genus, like HCoV-OC43 and HCoV-HKU1 (identified in 2005) [20]. These HCoVs regularly circulate within the human population, typically leading to benign infections, except in immunocompromised individuals, where outcomes can be more severe [21,22].

The emergence of more virulent coronaviruses, however, has underscored their significant epidemic potential. Severe Acute Respiratory Syndrome Coronavirus (SARS-CoV), a novel betacoronavirus, appeared in China in November 2002. It subsequently spread to 33 countries on five continents before subsiding in 2003, causing severe respiratory disease with a mortality rate of approximately 9% [23,24]. Nearly a decade later, another highly pathogenic betacoronavirus, Middle East Respiratory Syndrome Coronavirus (MERS-CoV), was identified. Infections first appeared in Saudi Arabia in 2012, and spread to 27 countries by 2018, causing severe respiratory illness with an even higher mortality rate than SARS-CoV. Most recently, SARS-CoV-2 emerged in China in late 2019, rapidly precipitating the global COVID-19 pandemic. As of May 2025, this crisis had resulted in over 7 million deaths worldwide that were attributed to COVID-19 and its related complications (https://data.who.int/dashboards/covid19/deaths, accessed on 22 May 2025). These successive coronavirus outbreaks have profoundly highlighted the critical need for rapid diagnostic capabilities for emerging pathogens, robust strategies for tracing viral origins, and the accelerated development of effective vaccines and therapeutics.

Coronavirinae, a subfamily of Coronaviridae of the order of Nidovirales, consists of some of the largest known RNA viruses, and virion diameters typically range from 80 to 120 nm, occasionally up to approximately 140 nm [20,21]. The single-stranded, positive-sense ribonucleic acid (RNA) genomes of these viruses replicate by utilizing the host cell’s machinery (i.e., ribosomes, Golgi apparatus, endoplasmic reticulum) for translation and assembly of viral proteins. Proteins that constitute the virion’s structure are Hemagglutinin-Esterase glycoprotein (HE), Spike (S), Envelope (E), Membrane (M), and Nucleoprotein (N). HE and S are surface proteins that enable attachment via receptors on the host cell surface. E and M proteins facilitate fusion for virion entry into the host cell. N packages genomic RNA into a nucleocapsid for incorporation, among other roles. However, SARS-CoV and SARS-CoV-2, being betacoronaviruses, do not contain HE [25,26]. S is the main surface protein by which betacoronaviruses bind to host cells. Non-structural proteins account for two-thirds of the genome, including the large RNA-dependent RNA polymerase (RdRP) and other proteins essential for replication and transcription. For a more detailed review of the coronavirus genome, see the referenced comprehensive works [14,21,27,28].

The global response to SARS-CoV spurred significant advancements in laboratory diagnostics for its era, with initial methods for detecting the virus being developed within weeks to months [29,30]. The MERS-CoV outbreak further accelerated research and development in coronavirus diagnostics, vaccine technology, and other biotechnological interventions aimed at controlling the spread of such fatal CoVs [31,32,33]. Research on SARS-CoV identified its spike (S) protein binding to the host cell’s angiotensin-converting enzyme-2 (ACE2) receptor as crucial for infection. This understanding led to the rapid identification of the SARS-CoV-2 S protein as a principal target for viral detection and variant tracking (via its mutations) during the COVID-19 pandemic [34,35,36,37,38]. Beyond the S protein, detection of the Nucleocapsid (N) or Envelope (E) proteins is also frequently utilized in various diagnostic assays [39,40,41].

**Figure 1:**
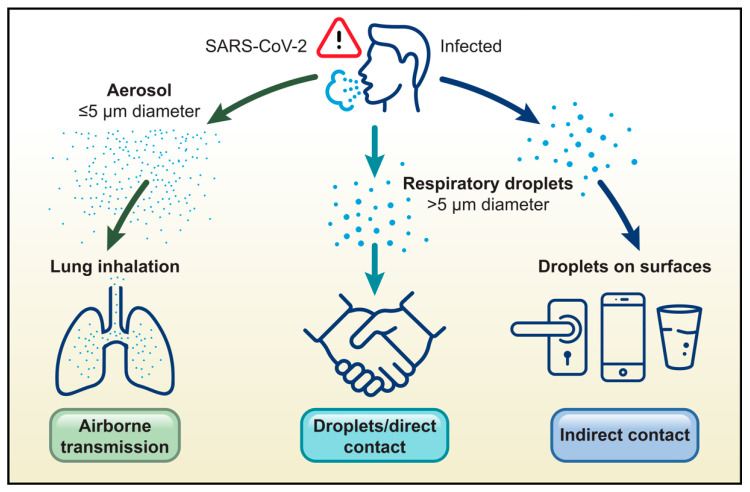
Modes of transmission of SARS-CoV-2. Airborne transmission is the inhalation of contaminated respiratory droplets aerosolized by the cough or sneeze of an infected individual, while other methods of transmission include direct contact or indirect contact with contaminated surfaces.

Epidemiological studies indicated that respiratory virus transmission commonly occurs through direct person-to-person contact, for instance, when infectious viral particles from droplet-contaminated hands are subsequently transferred to the recipient’s nose or eyes [23,41,42,43]. Extensive studies have been performed on virus transmissibility from fomites or droplets on surfaces, as well as aerosolization studies of defined particle size in various settings [43]. SARS-CoV-2 virion size is 100 nm in diameter on average, and infectious virus can be transmitted via aerosolization, when virus-containing droplets generally less than five microns in size have been aerosolized by the coughing or sneezing of an infected individual, or less frequently via indirect contact when larger respiratory droplets settle on commonly touched surfaces (Figure 1) [44,45,46].

**Figure 2:**
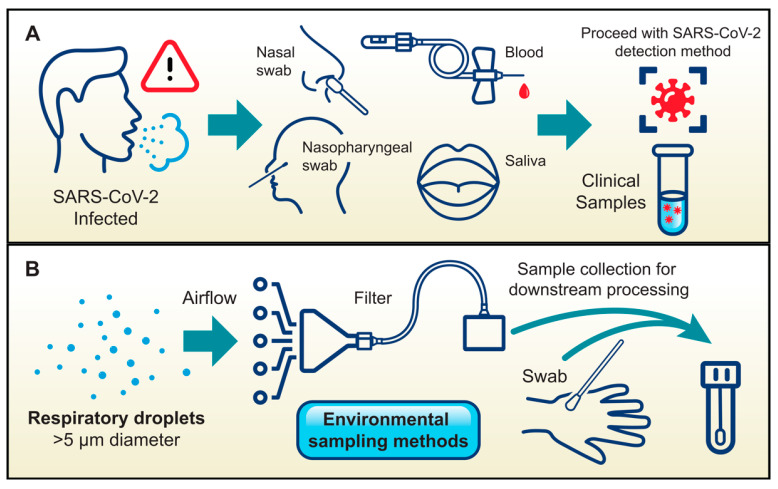
Sample collection methods for viral pathogen detection. (**A**) Common clinical samples for SARS-CoV-2 screening were obtained via nasal swab, nasopharyngeal swab, phlebotomy, or saliva collection. (**B**) Other environmental sampling methods that have been tested include the use of a filter or membrane from an air sampling unit or direct swabbing of hands or other surfaces.

Reflecting these transmission pathways, diverse sample collection methods were employed for SARS-CoV-2 detection during the COVID-19 pandemic. Clinically, common approaches included nasal swabbing, nasopharyngeal swabbing, and phlebotomy, while saliva-based detection, which is the least invasive and therefore a highly preferred method, was beta-tested (Figure 2A) [47,48,49,50,51]. Novel environmental sampling methods were also tested, where room air was collected via filtration or hands were swabbed. These samples were then processed clinically for the presence of SARS-CoV-2 (Figure 2B) [52].

Given this information, and considering the longevity of viral infectivity and incubation, detection methods for use during a similar future epidemic must possess the following characteristics: Real-time, Ease-of-collection, Affordable, Sensitive, Specific, User-friendly, Rapid, Equipment-free, Deliverable; or “REASSURED” as defined by the World Health Organization [53]. Additionally, optimal screening tests would detect and distinguish between any variant that could arise, and this feature is of great value for novel methods in development [54,55].

## 3. Advances in Viral Nucleic Acid Detection Methods

Numerous rapid methods for NA detection have been developed in response to the COVID-19 pandemic, some of which have the potential to become the next standard for infectious pathogen diagnosis. The continued struggle to overcome this hurdle of widespread implementation of novel screening methods deserves recognition, understanding that diagnoses are still predominantly assigned using decades-old technologies. The methodologies covered in this review are discussed with appreciation for the extensive scientific study over the past several decades that has enabled their development.

### 3.1. Necessity–the Mother of Invention

**Figure 3:**
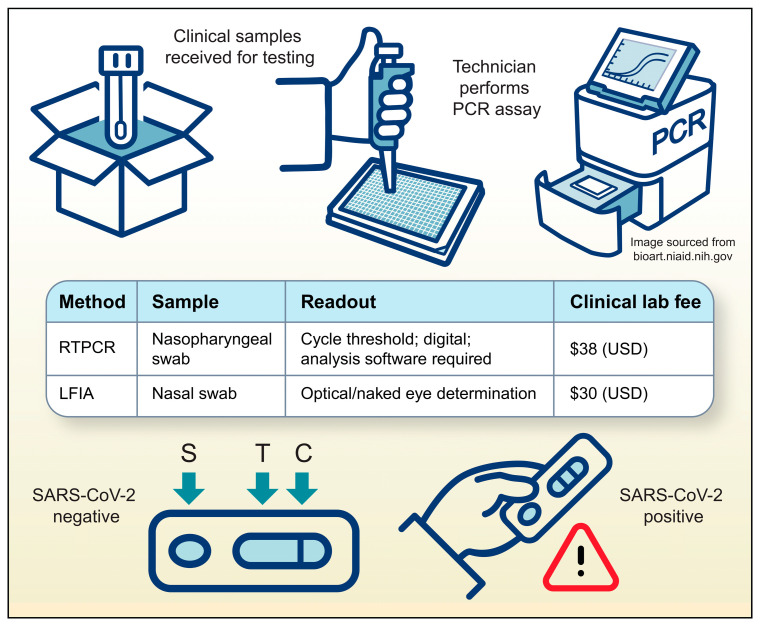
Comparison of the two most commonly deployed detection methods during the COVID pandemic. RTPCR is performed by trained technicians on specialized equipment, whereas LFIA is easily performed at POC by almost any user.

RT-PCR, which detects target RNAs and amplifies them into quantifiable cDNA fragments, has been the gold standard for diagnostic confirmation of viral infections for the last twenty years [29,56,57]. While traditional RT-PCR has well-documented limitations, the technology is continuously evolving to overcome these challenges through simplification, cost reduction, and reduced reliance on centralized laboratories. For instance, microfluidic and lab-on-a-chip platforms are enabling the development of smaller, faster, and more affordable PCR systems suitable for point-of-care (POC) settings. These miniaturized devices drastically reduce sample processing time and are designed for ease of use by non-specialized personnel [58,59]. Furthermore, techniques such as digital droplet PCR (ddPCR) significantly enhance diagnostic sensitivity by partitioning samples into thousands of nanoliter-sized droplets, allowing for the precise quantification of low-abundance nucleic acid targets [60]. Together, these innovations are expanding the utility of RT-PCR, making it a more accessible and powerful tool for infectious disease diagnostics, particularly in resource-limited or POC environments. Despite RT-PCR being the prevailing method for pathogen detection in clinics, the desire for rapid and accessible COVID-19 diagnostics led to the widespread use of the Lateral Flow ImmunoAssay (LFIA) for SARS-CoV-2 antigen detection. This simple, self-administered or POC detection method was widely marketed and made available to the general public in late 2020. However, LFIA false-negative and false-positive rates have shown the assay to be unreliable as a definitive diagnostic method [61,62]. Nevertheless, during the COVID-19 pandemic, these methods were extensively employed, contributing to an increase in Medicare Part B spending on lab tests by $1.3 billion [63]. Information from the American Medical Association and the Centers for Medicare and Medicaid Services Clinical Lab Fee Schedules from 2020 to 2022 can be used to approximate the cost of these clinical methods during the pandemic as $38 per RT-PCR and $30 per LFIA (Figure 3) [64,65,66].

As methods advance toward optimization, several novel approaches focus on Isothermal Amplification Methods (IAM) and Target Sensing Methods (TSM), which utilize more recent methodologies such as Reverse Transcriptase Loop-Mediated Isothermal Amplification (RT-LAMP), Rolling Circle Amplification (RCA), Clustered Regularly Interspaced Short Palindromic Repeat gene editing (CRISPR), and Aptamer-based biosensors (Aptasensors). The subsequent sections will review the methodology, advantages, and limitations of each of these techniques.

### 3.2. Isothermal Amplification Methods (IAM)

Isothermal amplification methods have emerged as a rapid and efficient alternative to conventional RT-PCR for SARS-CoV-2 detection, especially in POC settings [67]. These techniques amplify NAs at a constant temperature, eliminating the need for thermal cycling and complex equipment. This makes them well-suited for rapid diagnostics in diverse locations such as clinics, airports, and mobile testing units. Several isothermal amplification methods have been developed for SARS-CoV-2 detection, each with unique characteristics (Table 1). Here we summarize the mechanisms, advantages, and limitations of these amplification techniques, which include loop-mediated isothermal amplification (LAMP), recombinase polymerase amplification (RPA), nicking endonuclease amplification reaction (NEAR), rolling circle amplification (RCA), and NA sequence-based amplification (NASBA), highlighting their potential for rapid and accurate COVID-19 diagnosis.

#### 3.2.1. Loop-Mediated Isothermal Amplification (LAMP)

LAMP rapidly and cost-efficiently amplifies DNA with high specificity, making it suitable for detecting viral pathogens in resource-limited settings. Using a strand-displacing DNA polymerase, such as *Bst* polymerase, a set of four to six primers amplifies six to eight distinct regions of the target DNA. This primer set includes two inner primers (each containing two different sequences) and two outer primers. Some LAMP protocols also incorporate two-loop primers to enhance amplification efficiency [68]. Because SARS-CoV-2 is a single-stranded RNA virus, its detection via LAMP requires an initial conversion of viral RNA to complementary DNA (cDNA) using reverse transcriptase. This process, known as RT-LAMP, generates the DNA template necessary for LAMP [69,70,71]. LAMP creates a loop-based structure that allows for continuous amplification at a constant temperature (around 65 °C), eliminating the need for the thermal cycling required in traditional PCR. The resulting amplified product can be readily detected via fluorescent or colorimetric expression, enabling rapid and sensitive diagnosis of COVID-19 [72,73,74].

Several commercially available LAMP-based kits offer rapid and sensitive detection of SARS-CoV-2 RNA in patient samples. These assays can detect as few as one to ten copies of viral RNA per reaction, demonstrating a sensitivity comparable to the conventional RT-PCR methods [75,76,77]. However, some drawbacks with LAMP still need to be overcome. Designing effective primers for LAMP is more complex than for PCR, as multiple primers increase the risk of primer-dimer formation and non-specific amplification [78,79,80]. LAMP’s high sensitivity also increases the risk of contamination [81,82], and multiplexing (detecting multiple targets simultaneously) is more challenging than with PCR [80,83]. Furthermore, LAMP generates a complex mixture of DNA structures, complicating the analysis of amplicons for specific mutations or variants [84].

#### 3.2.2. Recombinase Polymerase Amplification (RPA)

RPA offers a streamlined approach to DNA amplification, utilizing enzymes to quickly initiate the reaction and enabling portable, POC, viral pathogen diagnostics. This method amplifies DNA or RNA targets with high sensitivity and specificity at a constant, low temperature, making it a compelling alternative to traditional PCR-based methods, especially in resource-limited settings [85]. For SARS-CoV-2 detection, an initial reverse transcription step converts the viral RNA into cDNA. RPA then utilizes two key enzymes: a recombinase (typically *UvsX*) and a strand-displacing polymerase (typically *Bsu*). The recombinase, combined with primers and assisted by ATP and polyethylene glycol, forms a complex that locates and binds to the target DNA sequence. This initiates strand displacement, allowing the polymerase to bind and synthesize a new complementary strand. A single-stranded DNA-binding protein (SSB) further aids the process by stabilizing the displaced strands. This cycle repeats, resulting in rapid exponential amplification of the target sequence. The entire RPA reaction occurs quickly, often producing detectable amplification products within 20 min. Its isothermal nature, operating at a constant temperature between 37 °C and 42 °C, eliminates the need for thermal cycling equipment, enhancing portability for field applications [86].

However, RPA can be more prone to non-specific amplification, often requiring secondary amplification and detection steps, which adds complexity [87,88]. The lack of specialized software for RPA primer design can lead to increased costs and time for primer development [89,90]. Finally, while RPA often requires less expensive equipment, the reagents themselves can be more costly, potentially impacting cost-effectiveness for large-scale testing [90].

#### 3.2.3. Nicking Endonuclease Amplification Reaction (NEAR)

NEAR provides a highly sensitive method for detecting even trace amounts of viral NAs, leveraging a unique nicking mechanism for precise amplification. Two main enzymes are used in NEAR amplification: a nicking endonuclease (responsible for cleaving DNA at specific recognition sites) and a strand-displacing DNA polymerase. The reaction occurs at a constant temperature, typically between 55 °C and 59 °C. A diverse array of commercially available nicking endonucleases operates across a broad temperature spectrum, each recognizing a unique sequence. Unlike PCR, NEAR utilizes specialized primers with a distinct structure. These primers feature a stabilizing region at the 5′ end, a target-specific region at the 3′ end, and a centrally located nicking endonuclease recognition site. The reaction initiates with the 3′ end of the primer hybridizing to the target sequence [91,92]. In the context of SARS-CoV-2 detection, NEAR offers the advantage of bypassing the reverse transcription step in some protocols [91]. Primers directly hybridize with the target RNA, and the DNA polymerase extends the primer, forming a DNA/RNA hybrid. Subsequently, the nicking enzyme introduces a nick in the newly synthesized DNA strand, and the polymerase extends from this nick, displacing the downstream strand. Exponential amplification is achieved through repeated nicking, extension, and displacement cycles, enabling rapid target sequence detection, often within 5–10 min [93].

While NEAR offers several advantages for NA detection, it also has some limitations. NEARs can sometimes produce non-specific amplification products, potentially leading to false-positive results. This issue can arise from primer dimer formation, mispriming, or non-specific nicking endonuclease activity [91]. Designing effective NEAR primers can be more complex than PCR primers, as they require specific features such as a nicking enzyme recognition site and a stabilizing region, which must be carefully considered during the design process [91,94]. Furthermore, the reaction relies on the precise interplay between nicking enzymes and strand-displacing DNA polymerases. This dependency on specific enzyme activities can make the reaction sensitive to variations in reaction conditions, potentially affecting the reproducibility and reliability of the assay [91].

#### 3.2.4. Rolling Circle Amplification (RCA)

RCA was developed in the early 1990s and has since found diverse applications in various fields, including the detection of SARS-CoV-2 [95]. Among the different RCA techniques, padlock probe RCA has emerged as the most widely used method for SARS-CoV-2 detection due to its high specificity and sensitivity. With padlock probe RCA, a specially designed single-stranded DNA probe, called a “padlock probe,” plays a crucial role in initiating the amplification process. This probe has two segments (“arms”) at its ends that are complementary to a specific region of the SARS-CoV-2 RNA genome. A linker region connects these target-specific arms. When the padlock probe encounters the target RNA, the two arms bind to their complementary sequences, bringing the ends of the probe close together. An enzyme called ligase then joins the two ends of the probe, forming a closed circular DNA molecule. This circularized padlock probe serves as the template for RCA, where a strand-displacing polymerase initiates DNA synthesis (Phi29 DNA polymerase), generating a long DNA molecule containing multiple copies of the circular probe sequence [96]. Advanced RCA techniques like multi-padlock RCA, employing multiple probes targeting different genomic regions, have been developed to further enhance sensitivity and detect mutated strains [97]. Limitations of RCA are related to design, cost and specificity. Padlock probes are typically close to 100 base pairs in length, which are a challenge to design and optimize. Factors such as probe length, melting temperature, and enzyme concentrations require careful consideration to ensure efficient and specific amplification. Additionally, the cost of probes and enzymes can be relatively high compared to other amplification techniques. Another limitation is the potential for background interference during signal detection, which can reduce the accuracy and specificity of results, especially in complex samples or when detecting low-abundance targets. Non-specific amplification can also occur, even with padlock probes, due to factors like primer dimer formation or cross-reactivity with non-target sequences. This can lead to false-positive results, particularly with complex templates or very low template concentrations, even under optimized conditions [97,98].

#### 3.2.5. Nucleic Acid Sequence-Based Amplification (NASBA)

NASBA is a molecular biology technique used to amplify single-stranded RNA, making it well-suited for applications such as gene expression analysis, cancer diagnostics, and infectious disease detection, including the detection of SARS-CoV-2. This isothermal method operates at a constant temperature, typically 41 °C, thereby simplifying the procedure compared to techniques that require thermal cycling. NASBA relies on three key enzymes: reverse transcriptase (RT), RNase H, and T7 RNA polymerase. Two specifically designed primers are crucial to the process. One primer contains a T7 RNA polymerase promoter sequence at its 5′ end and a target-specific sequence at its 3′ end. This primer binds to the target RNA, and RT generates a cDNA strand. Following cDNA synthesis, RNase H degrades the original RNA template, leaving the cDNA. The second primer then binds to this cDNA, and RT synthesizes a second DNA strand, creating a double-stranded DNA molecule that incorporates the T7 promoter. T7 RNA polymerase recognizes this promoter and transcribes multiple RNA copies from the DNA template. These newly synthesized RNA molecules can then serve as templates for further amplification, leading to exponential increases in the target RNA sequence [99,100]. NASBA exhibits high sensitivity, capable of detecting as few as 100–200 copies of SARS-CoV-2 viral RNA per milliliter of sample. The entire NASBA reaction typically takes 60 to 90 min to complete [101].

Despite its advantages, NASBA faces several limitations. The technique requires expensive commercial kits and reagents, and the use of three different enzymes increases overall costs and makes optimization challenging. The need for specific enzymes with particular functions limits NASBA’s flexibility compared to techniques like PCR [102]. Balancing multiple enzymes in the reaction can be difficult, affecting reproducibility and reliability in clinical settings [103]. Furthermore, NASBA is susceptible to false positives, necessitating additional probe-specific detection methods, which can further increase costs [104]. These limitations have hindered NASBA’s widespread adoption compared to other NA amplification techniques, despite its high sensitivity and specificity for RNA detection.

### 3.3. Target Sensing Methods (TSM)

**Figure 4:**
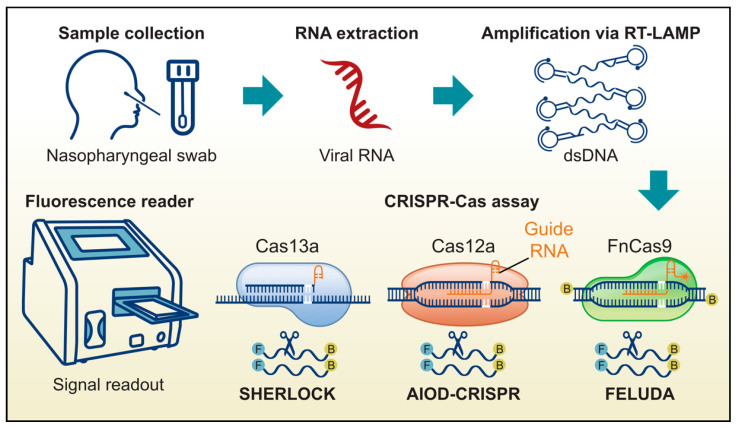
The CRISPR-based workflow for pathogen detection. The process begins with sample collection and NA extraction. Viral RNA is first amplified, often using an isothermal method, such as Reverse Transcriptase Loop-Mediated Isothermal Amplification (RT-LAMP). The amplified product is then used as the template in a CRISPR-Cas assay. In this step, a guide RNA (gRNA) directs the Cas enzyme to a specific target on the virus. Upon binding, the Cas enzyme is activated and exhibits collateral cleavage activity, severing nearby reporter molecules. This cleavage separates a fluorophore from its quencher, generating a fluorescent signal that can be measured with a microplate reader to indicate the presence of the pathogen.

The development of isothermal amplification methods represented a significant step forward in SARS-CoV-2 detection, offering improvements in speed and cost-effectiveness. However, the persistent challenge of minimizing false-positive results prompted the exploration of more targeted methods, such as CRISPR-based techniques. Several such methods, including SHERLOCK (Specific High Sensitivity Enzymatic Reporter unLOCKing), AIOD-CRISPR (All-In-One Dual CRISPR-Cas12a), DETECTR (DNA Endonuclease Targeted CRISPR Trans Reporter), and FELUDA (FnCAS9 Editor Linked Uniform Detection Assay), have been developed to enhance both the sensitivity and specificity of viral detection. The integration of these CRISPR-based assays with isothermal amplification techniques, such as LAMP, RCA, or RPA, has further contributed to improvements in the accuracy of SARS-CoV-2 diagnostics (Figure 4) [105]. Additionally, biosensors, such as electrochemical sensors and aptasensors, have been developed to improve assay sensitivity and specificity [106,107]. Here, we highlight several advantages and limitations of these target sensing methods in SARS-CoV-2 detection.

#### 3.3.1. Specific High Sensitivity Enzymatic Reporter Unlocking (SHERLOCK)

SHERLOCK employs the CRISPR-Cas13 system, where a guide RNA, designed to be complementary to a specific SARS-CoV-2 RNA sequence, directs the Cas13 enzyme to the viral RNA target. In SARS-CoV-2 detection, samples are typically pre-amplified using RT-LAMP or RT-RCA, followed by in vitro transcription using T7 RNA polymerase. This amplification generates numerous copies of the target RNA, enhancing detection sensitivity. If viral RNA is present, Cas13 binds to the target RNA and becomes activated, triggering collateral cleavage of any RNA present. SHERLOCK assays also include reporter RNA molecules linked to a signal-generating substance (e.g., a fluorescent molecule) that is normally quenched. Upon Cas13 activation and subsequent RNA cleavage, these reporter molecules are cleaved, releasing the signal and indicating the presence of SARS-CoV-2 RNA [108,109].

While SHERLOCK demonstrates significant potential for SARS-CoV-2 detection, several limitations warrant consideration. Although SHERLOCK exhibits high sensitivity, it is constrained by a definitive limit of detection (LOD). Studies have reported varying LODs, ranging from 6.75 copies per microliter of viral transport medium to approximately 42 copies per reaction. Consequently, samples containing very low concentrations of viral RNA, particularly during early infection stages or in individuals with low viral loads, may escape detection, potentially resulting in false-negative results for samples near the LOD threshold [110]. The RNA-based nature of SHERLOCK renders it susceptible to RNase interference, which can compromise test accuracy. RNase contamination may lead to false negatives due to RNA degradation or false positives if carried over to the CRISPR-Cas detection phase [110,111]. While SHERLOCK offers multiplexing capabilities for simultaneous detection of multiple targets, this feature introduces additional assay complexity and may necessitate further optimization [112]. Furthermore, SHERLOCK is primarily designed for qualitative SARS-CoV-2 detection, potentially limiting its ability to provide precise viral load quantification. These limitations underscore the need for ongoing refinement and validation of SHERLOCK-based assays to enhance their performance and broaden their applicability in diverse clinical settings.

#### 3.3.2. DNA Endonuclease Targeted CRISPR Trans Reporter (DETECTR) and All-In-One Dual CRISPR-Cas12a (AIOD-CRISPR)

DETECTR and AIOD-CRISPR use the CRISPR-Cas12a system for SARS-CoV-2 detection in a streamlined, single-tube format. Following RNA extraction and isothermal amplification (e.g., RT-LAMP or RT-RPA with reverse transcriptase) of patient samples, DETECTR utilizes one crRNA, while AIOD-CRISPR utilizes two different crRNAs to guide Cas12a, thereby enhancing the sensitivity and specificity of SARS-CoV-2 RNA detection. The reaction tube contains all necessary components, including Cas12a. Upon target binding, Cas12a is activated, initiating collateral cleavage of a fluorophore-quencher-labeled ssDNA probe. This cleavage releases the fluorophore, generating a detectable signal that indicates the presence of SARS-CoV-2 RNA. While Cas12a typically requires a protospacer adjacent motif (PAM) for target recognition and cleavage, recent advancements have overcome this limitation. PAM-less strategies have been developed, either by introducing PAM sequences into primers for isothermal amplification or by designing crRNAs adjacent to primer recognition sites in the target sequence. These strategies have removed limitations on crRNA design and enhanced the sensitivity of SARS-CoV-2 detection [105,113].

AIOD-CRISPR demonstrates higher sensitivity than DETECTR, with the ability to detect as few as 4.6 copies of SARS-CoV-2 N RNA targets in 40 min. The dual crRNA approach in AIOD-CRISPR contributes to its improved sensitivity and specificity [113]. However, this increased complexity may make designing, implementing, and optimizing the assay more challenging [114]. Despite these limitations, both methods offer rapid, sensitive, and specific detection of SARS-CoV-2, with potential for POC applications.

#### 3.3.3. FnCAS9 Editor Linked Uniform Detection Assay (FELUDA)

FELUDA is a CRISPR-Cas9-based diagnostic platform using a Cas9 ortholog from *Francisella novicida* (FnCas9). FELUDA reveals high specificity, sensitivity, and minimal off-target activity, making it highly effective for SARS-CoV-2 detection [115,116]. The process begins with isolating RNA from patient samples, which is then converted into cDNA through reverse transcription. The cDNA undergoes amplification via methods like PCR or RPA using biotin-labeled primers. The assay used the FnCas9 enzyme, complexed with a gRNA specifically designed to target particular SARS-CoV-2 sequences. The amplified product is mixed with the FnCas9-gRNA complex labeled with fluorescein amidite (FAM). If the target sequence is present, the complex binds to the biotin-labeled DNA. This mixture is applied to a paper strip embedded with gold nanoparticles conjugated with anti-FAM antibodies. The lateral flow mechanism allows the complex to migrate up the strip. A visible test line appears if the target sequence is present, while a control line ensures the assay’s validity. FELUDA provides rapid and accurate results comparable to qRT-PCR assays, with sensitivity and specificity exceeding 95%. Its low cost and ease of use make it particularly suitable for resource-limited settings and large-scale testing efforts [115,116,117].

FELUDA’s high specificity makes it susceptible to mutations in key target regions, especially those near the PAM. Such mutations (PAM-proximal or PAM-distal) can disrupt FnCas9 binding and cleavage, potentially causing false negatives when detecting SARS-CoV-2 variants [117].

#### 3.3.4. Aptamer-Based Biosensors (Aptasensors)

An aptamer is a short, single-stranded nucleic acid (DNA or RNA) or, less commonly, a peptide that folds into a unique three-dimensional structure. This structural conformation enables the aptamer to bind specific target molecules—such as proteins, small molecules, or even cells—with high affinity and specificity, functioning much like a chemical antibody to detect or inhibit its target. In contrast, a guide RNA (gRNA) in CRISPR systems does not act by folding into a complex structure for molecular recognition. Instead, its primary function is to direct a Cas enzyme to a precise DNA or RNA sequence, where the enzyme mediates cleavage or other modifications. The gRNA achieves this by containing a ~20-nucleotide “spacer” region that is complementary to the target sequence, allowing recognition through straightforward Watson–Crick base pairing. While aptamers rely on tertiary structure-driven molecular recognition, gRNAs operate mainly through sequence-specific hybridization [50,118,119]. Aptamers are employed in assays using various detection techniques, such as optical and electrochemical, including novel field-effect transistors, and aptamer-based assays have been commercially available for veterinary sample testing for more than ten years [120,121]. In recent years, aptamer-based assays have emerged as powerful tools for clinical diagnostics and disease monitoring across several major fields. In oncology, for instance, these assays are used to detect key biomarkers such as prostate-specific antigen (PSA), cancer-related exosomes, and circulating tumor cells (CTCs) [122,123]. Similarly, for neurological disorders, aptamer-based assays have been developed to detect indicators of Alzheimer’s and Parkinson’s disease, including amyloid-beta and tau proteins [124]. Pushing the technological frontier, recent innovations include wearable, field-effect transistor (FET) based aptasensors for the noninvasive, real-time monitoring of hormones like cortisol and estradiol [125,126]. Aptasensors show great promise for developing superior detection methods for viral pathogens, primarily due to the distinct advantages aptamers offer over traditional antibodies as recognition elements [127,128]. A key benefit is their superior chemical and thermal stability; unlike protein-based antibodies that often require a cold chain for storage and transport, nucleic acid aptamers are robust across a wide range of temperatures and pH conditions. This makes them ideal for point-of-care tests intended for resource-limited or field settings [129,130]. Furthermore, as aptamers are produced via chemical synthesis rather than biological processes, they exhibit virtually no batch-to-batch variability, ensuring high reproducibility and consistency in sensor performance. Their synthetic nature also allows for easy and precise site-specific modification with reporter molecules (e.g., fluorophores, redox labels), facilitating the design of advanced sensing mechanisms that are more challenging to achieve with antibodies [131]. Finally, their small size (~10–20 kDa compared to ~150 kDa for an IgG) allows for denser immobilization on sensor surfaces and can provide better access to binding sites on the viral target that may be sterically hindered from a larger antibody [130].

Furthermore, multiple novel target-sensing methods (TSM) could be combined to produce assays with even greater specificity and sensitivity. To overcome the challenge of low target concentrations in raw samples like blood or saliva, a two-stage strategy is typically employed. First, a nucleic acid amplification technique—often an isothermal method like RCA, LAMP, RPA, or NASBA—is used to generate a high copy number of the target sequence. Second, an electrochemical biosensor serves as a low-cost and portable alternative to expensive optical systems for detection. This sensor transduces the presence of the amplified product (amplicons) into a quantifiable electrical signal, such as a change in current, voltage, or impedance. For example, an ultra-sensitive electrochemical biosensor using RCA can detect as low as one copy/μL of SARS-CoV-2 RNA by hybridizing RCA amplicons with redox-active labeled probes [132]. A recent study has explored combining CRISPR-Cas13a directly with electrochemical biosensors in an amplification-free format. In this approach, a pre-formed Cas13a-crRNA complex recognizes and binds to the target SARS-CoV-2 RNA. This binding event activates the potent “trans-cleavage” (or collateral RNase) activity of Cas13a, causing it to indiscriminately cleave nearby reporter RNA (reRNA) molecules that are coupled to the electrode surface. As these reporters are cleaved, attached redox molecules are released, generating a measurable change in the electrochemical current [133]. However, because this method lacks a pre-amplification step, its sensitivity is inherently limited. It performs poorly when detecting the low viral RNA concentrations typical of early-stage infections, especially compared to established CRISPR diagnostics like SHERLOCK and DETECTR, which incorporate amplification [133]. Therefore, integrating a nucleic acid amplification stage with a CRISPR-electrochemical system is a critical strategy for achieving the ultra-high sensitivity required for the early detection of infectious agents. This synergistic approach provides several key advantages, including enhanced sensitivity, portability, and rapid response times. The quantitative nature of the signal enables the precise measurement and quantification of nucleic acid concentrations, critical for applications like viral load estimation. However, these benefits are balanced by significant practical challenges. Combining these technologies increases assay complexity and the risk of cross-contamination from amplified products. Furthermore, this integration requires meticulous optimization to prevent false signals and can increase device cost, potentially complicating portability compared to simpler sensor formats [134,135,136].

Additionally, recent advances in the Systematic Evolution of Ligands by Exponential Enrichment (SELEX) process, such as improved separation methods and in silico predictions of aptamer-target interactions enabled by artificial intelligence (AI) [53,137,138], ably shortened the time required to identify suitable aptamers for diagnostic applications. However, while these technological improvements accelerate aptamer selection and enhance affinity or specificity, they do not, by themselves, guarantee that all subsequent diagnostic platforms incorporating these aptamers will fulfill all REASSURED criteria. The ultimate diagnostic performance also depends on the design and integration of amplification probes, the sensing platform, and assay development steps. Therefore, parallel advances in amplification probe design, which form the core of many molecular diagnostics—including those discussed in this review—must also be considered. Although the development costs of aptamer-based assays may remain higher than those of some competing approaches owing to the iterative selection and sequence verification required in SELEX, an optimized aptasensor platform has the potential for substantial cost savings and rapid deployment at scale during future infectious disease outbreaks.

## 4. Discussion

Development efforts for improved viral pathogen detection methods during the COVID-19 pandemic expedited the implementation of new testing methods for coronaviruses within days to weeks, in part thanks to the NIH’s Rapid Acceleration of Diagnostics (RADx) initiative of 2020 [9,139]. Since conventional biomarker quantification methods such as Mass Spectrometry (MS), RT-PCR, and Flow Cytometry are costly and require specialized equipment and highly trained staff [140,141], novel methods to be deployed for future pandemics should fit the REASSURED criteria. The COVID-19 pandemic spurred the development of numerous rapid NA detection assays that utilize methodologies extending beyond traditional lab-based PCR. For instance, integrated platforms like Cepheid’s Xpert Xpress and the ARIES system employ rapid, automated real-time PCR. Other approaches use isothermal amplification, such as the Abbott ID NOW platform. A major innovation was the application of CRISPR-based diagnostics, exemplified by the DETECTR platform which utilizes CRISPR-Cas12 for highly specific target recognition. Concurrently, extensive research also focused on other novel methods, including assays based on Reverse Transcriptase Loop-Mediated Isothermal Amplification (RT-LAMP) and aptamer-based biosensors (aptasensors), further broadening the landscape of diagnostic possibilities. Point-of-care PCR testing has been realized [142], and continued progress is being made on reducing the time-to-result [143]. Isothermal amplification methods, such as RCA or LAMP, along with target sensing methods, such as CRISPR-Cas or Aptasensors, will reduce the costs of future pandemic response [144].

In addition to the aforementioned isothermal amplification and target sensing approaches, the field of microfluidics has become a cornerstone in the development of miniaturized diagnostic assays for acute respiratory tract infections (ARTIs), including COVID-19. Microfluidic platforms enable the manipulation of small fluid volumes, resulting in assays with minimal sample and reagent requirements, faster reaction kinetics, and shortened turnaround times compared to conventional methodologies. Recent advances have led to the integration of microfluidic chips with isothermal amplification techniques such as RT-LAMP and RT-RPA, facilitating on-chip sample processing, nucleic acid extraction, amplification, and real-time detection in a closed, user-friendly format. Furthermore, microfluidic-based immunoassay systems have demonstrated the ability to rapidly identify viral antigens or host antibodies with high sensitivity, supporting both early detection and serological surveillance. These compact, automated platforms are particularly advantageous for point-of-care (POC) testing and deployment in resource-limited settings, thereby enhancing diagnostic capacity during outbreaks. A comprehensive overview of recent innovations and their application for ARTIs, including SARS-CoV-2 diagnostics, is provided in the review by Liu et al. [145].

### 4.1. Complexities of Implementation

In our quest for pandemic preparedness regarding pathogen identification in the population, we must bear in mind that, while the detection of viral pathogens in clinical samples has long been a method for identifying infections, detection alone is insufficient for disease prediction. Widespread detection measures have historically been unreliable in accurately depicting disease outcomes, as mutation and attenuation play essential roles in the knockdown of a pathogen. However, within the past decade, progress has been made due to newly available technologies related to epidemiological data modeling and AI [146,147,148,149]. Qualitative results are problematic due to technical reproducibility and lack of rigor; for example, RT-PCR cycle thresholds (Ct) are not an objective standard across clinics. Viral NAs may be present at varying gradations across specimens regardless of the presence of disease due to the circulation of residual copied DNA (e.g., inconsistently loaded) or due to preexisting immunity. Additionally, universal testing adds unnecessary costs and labor burdens, as studies have shown that up to 30% of lab tests are unnecessarily repeated, overused, or inappropriately prescribed [4,150,151,152].

Throughout the COVID-19 pandemic, the transmission rates and mechanisms of SARS-CoV-2 infection have been extensively studied, particularly in clinical settings. Johns Hopkins University’s Center for Systems Science and Engineering (CSSE) provided as close to a real-time global update on reported cases as had ever been seen before [153]. The CSSE COVID-19 Dashboard was tallying counts semi-automatically from February 2020 through March 2023. This achievement for clinical data tracking and compilation was monumental, even though these counts were not entirely accurate based on publicly available data from CDC Wonder, where cause of death “from COVID-19” versus “with COVID-19” can be delineated [154]. Nevertheless, the visual aid for tracking coronavirus infection was constantly highlighted in mainstream media during that time and served to perpetuate research efforts related to transmission, as mentioned previously.

As more advanced methods of screening for viral pathogens are sought, we should bear in mind that widespread testing for the presence of a virus may be unwarranted if virulence is low. Infectivity, or contagiousness, does not equate to virulence of the organism, which is commonly misunderstood by the general public. This misunderstanding about contagions often leads to undue anxiety about transmission regardless of virulence, and costly measures may be unnecessarily implemented. The level of response to emerging viral pathogens should correlate with virulence, the organism’s ability to cause severe disease. Additionally, attenuation of virulence due to naturally occurring deleterious mutations is the normal process for the lifespan of viral pathogens [55]. While public health messaging has emphasized transmissibility, some analyses have questioned the value of repeated screenings for previously infected individuals. From a public health perspective, the allocation of resources for broad testing and vaccination strategies requires ongoing evaluation to ensure a clear and direct benefit to at-risk populations [155,156].

### 4.2. Cost of Implementation

Routine testing for endemic viruses that typically cause mild, self-limiting illnesses like the common cold has not been a standard clinical practice, largely due to a perceived unfavorable cost-to-benefit ratio for widespread screening [157,158]. However, the speed of modern-day travel has increased the probability that viral pathogens, which would otherwise remain a local hazard, can quickly become a global pandemic, nearly impossible to restrict [23,159]. Therefore, some level of judicious clinical testing is necessary, and the cost should be considered wisely.

**Figure 5:**
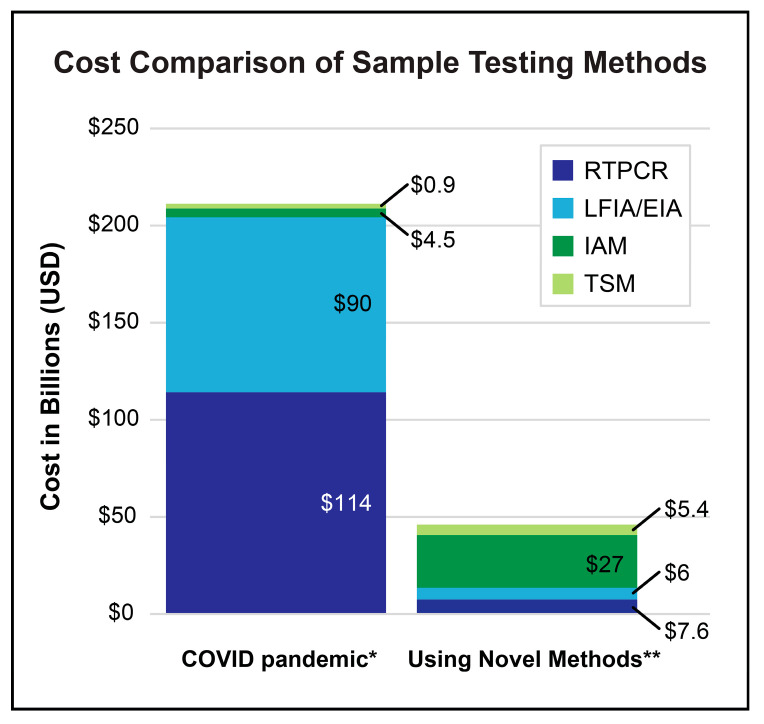
Cost of the most utilized SARS-CoV-2 screening methods during the COVID-19 pandemic in comparison to the cost of the same number of tests if novel methods replace existing methods. * Pandemic estimate of clinical use of RT-PCR, and LFIA plus EIA = USD 204 billion; and 10% beta-testing of IAM and TSM = USD 5.4 billion. ** By replacing 80% of today’s standard clinical tests with novel methods, RT-PCR and LFIA plus EIA reduce to USD 13.6 billion, while IAM and TSM increase to only USD 32.4 billion.

While evaluation of pandemic-associated financial costs is typically focused on excess expenditures, it is important to note that most clinical settings suffer from lost revenue and lost labor [160]. However, for our limited analysis of costs related to widespread testing of clinical samples based on information obtained from Centers for Medicare & Medicaid Services (CMS), American Medical Association (AMA), and other reports, comparisons can be made to determine hypothetical cost-savings from implementation of novel methods [64,65,161,162]. If the costs of COVID-19 testing in the United States during the COVID-19 pandemic (2020 through 2022) could be estimated at approximately USD 209.4 billion, then the cost for an equivalent number of tests using novel methods in place of RT-PCR and LFIA plus EIA could potentially have been reduced to USD 46 billion (Figure 5). This illustration exemplifies the potential economic benefit of novel coronavirus testing methods after optimization.

As seen with COVID-19, Long COVID or post-acute sequelae of SARS-CoV-2 (PASC), and mRNA-generated Spike protein pathogenicity, complications can be short or long-term and include autoimmune, cardiovascular, neurological and potential oncological effects [163,164,165,166]. Currently, in vivo methods for rapid identification of viral pathogen infection are too invasive and cumbersome. Costly techniques for in vivo detection based on symptomology, such as MRI or PET/SPECT that require intravenous injection, which is not advised during viral infection, have no preventative value, but only serve to identify post-infection damage. The innovative developments mentioned in this review forecast novel strategies for early detection of COVID-19 or Spikeopathy sequelae and their potential mitigation. Ideally, in vivo detection in real time could be visualized to predict impact and outcomes on health. Hypothetically, Aptasensors or a CRISPR-Cas assay could be designed to detect protein aggregates or viral NAs in the CNS during infection, perhaps correlated to the presence of viral load or inflammatory cytokine levels due to a leaky blood–brain barrier. A REASSURED detect-and-treat method for viral pathogens is conceivable as bionanotech continues to develop.

## 5. Conclusions

Knowing that coronaviruses are endemic and highly transmissible with the potential to cause severe disease, continuous improvement in methods for their molecular detection is a worthwhile cause in preparation for future outbreaks. The techniques discussed in this review offer significant potential for addressing future pandemics more effectively, with emphasis on the importance of specificity, accuracy, availability, and cost-efficiency in diagnostic methods. During the initial stages of the COVID-19 pandemic, RT-PCR served as the primary diagnostic tool. However, the overwhelming number of samples strained laboratory capacities, ultimately reducing the speed of SARS-CoV-2 detection, particularly at point-of-care locations that lacked adequate equipment or trained technicians. In comparing the two most widely deployed tests for SARS-CoV-2 detection, LFIA appears to have been more consumer-oriented and profit-motivated, offering an enticing rapid readout at a comparable cost to RT-PCR, albeit with lower reliability and validity. To produce truly improved detection methods for the next pathogen(s) of pandemic potential, ideal POC devices will epitomize the acronym REASSURED (Real-time, Ease-of-collection, Affordable, Sensitive, Specific, User-friendly, Rapid, Equipment-free, Deliverable). In this context, isothermal amplification combined with CRISPR-based methods and biosensors emerges as a diverse toolkit for rapid response to new viral variants. These advanced technologies offer rapid detection capabilities, with CRISPR-based assays providing results in under 40 min and demonstrating high sensitivity and specificity comparable to traditional RT-PCR methods. Likewise, aptamer-based biosensor assays can now detect infectious pathogens in as little as five minutes with improved cost-effectiveness and employability compared to RT-PCR. Furthermore, these newer methods can be quickly adapted to identify emerging variants, effectively addressing the challenge of false negatives associated with viral mutations. Notably, the sensitivity of several methods has now advanced to the point that LOD is achievable at attogram levels. [8,53,108,141,144,167]. Some of these techniques are being developed into portable and user-friendly devices suitable for decentralized testing, promoting convenience while allowing for simultaneous detection of multiple pathogens or variants. When employed by public health systems, these innovative diagnostic tools can facilitate rapid transition to treatment strategies, reducing the burden of hospitalization due to delayed treatment. These improvements in detection protocols and available rapid screening methods are not trivial and will undoubtedly have significant impacts on the handling of clinical responses to viral pathogens, ultimately strengthening our preparedness for future pandemics.

## Figures and Tables

**Table 1:** Characteristics of primary isothermal amplification methods.

Method.	Template	Key Enzymes	Primer(s)	Temp. (°C)	Main Features
LAMP	DNA or RNA	Bst DNA polymerase (± RT)	4–6	60–65	High specificity, loop structures, turbidimetric/colorimetric detection
RPA	DNA	Recombinase, SSB, strand-displacing DNA polymerase	2	37–42	Rapid (20–40min), low temperature, portable
NEAR	DNA	strand-displacing DNA polymerase, nicking endonuclease	2	55–59	Ultra-rapid, uses endonuclease for nicking, suitable for point-of-care
RCA	Circular DNA	Ligase, Phi29 DNA polymerase	1	30–42	Long concatemer products, simple design, high yield
NASBA	RNA	Reverse transcriptase, T7 RNA polymerase, RNase H	2	40–41	RNA as the main target, transcription-based, isothermal

## Data Availability

The raw data supporting the conclusions of this article will be made available by the authors on request.

## Abbreviations

The following abbreviations are used in this manuscript:

AOID-CRISPR	All-in-One-CRISPR
CoV	Coronavirus
COVID-19	Coronavirus Infectious Disease-2019
CRISPR	Clustered Regularly Interspaced Short Palindromic Repeats
DETTECTR	DNA Endonuclease Targeted CRISPR Trans Reporter
EIA	Enzymatic ImmunoAssay
FELUDA	FnCAS9 Editor Linked Uniform Detection Assay
IAM	Isothermal Amplification Methods
LAMP	Loop-Mediated Isothermal Amplification
LFIA	Lateral Flow Immunoassay
MERS	Middle East Respiratory Syndrome
MRI	Magnetic Resonance Imaging
NA	Nucleic Acid
NASBA	Nucleic Acid Sequence-Based Amplification
NEAR	Nicking Endonuclease Amplification Reaction
PET/SPECT	Positron Emission Tomography/Single Photon Emission Computed Tomography
POC	Point-of-Care
RCA	Rolling Circle Amplification
RNA	Ribonucleic Acid
RPA	Recombinase Polymerase Amplification
RTPCR	Real-Time reverse transcriptase Polymerase Chain Reaction
SARS	Severe Acute Respiratory Syndrome
SHERLOCK	Specific High Sensitivity Enzymatic Reporter Unlocking
TSM	Target Sensing Methods
